# M1CR0B1AL1Z3R—a user-friendly web server for the analysis of large-scale microbial genomics data

**DOI:** 10.1093/nar/gkz423

**Published:** 2019-05-22

**Authors:** Oren Avram, Dana Rapoport, Shir Portugez, Tal Pupko

**Affiliations:** The School of Molecular Cell Biology & Biotechnology, George S. Wise Faculty of Life Sciences, Tel Aviv University, Tel Aviv 69978, Israel

## Abstract

Large-scale mining and analysis of bacterial datasets contribute to the comprehensive characterization of complex microbial dynamics within a microbiome and among different bacterial strains, e.g., during disease outbreaks. The study of large-scale bacterial evolutionary dynamics poses many challenges. These include data-mining steps, such as gene annotation, ortholog detection, sequence alignment and phylogeny reconstruction. These steps require the use of multiple bioinformatics tools and ad-hoc programming scripts, making the entire process cumbersome, tedious and error-prone due to manual handling. This motivated us to develop the M1CR0B1AL1Z3R web server, a ‘one-stop shop’ for conducting microbial genomics data analyses via a simple graphical user interface. Some of the features implemented in M1CR0B1AL1Z3R are: (i) extracting putative open reading frames and comparative genomics analysis of gene content; (ii) extracting orthologous sets and analyzing their size distribution; (iii) analyzing gene presence–absence patterns; (iv) reconstructing a phylogenetic tree based on the extracted orthologous set; (v) inferring GC-content variation among lineages. M1CR0B1AL1Z3R facilitates the mining and analysis of dozens of bacterial genomes using advanced techniques, with the click of a button. M1CR0B1AL1Z3R is freely available at https://microbializer.tau.ac.il/.

## INTRODUCTION

In a typical microbial genomics study, a few dozen bacterial samples are sequenced using next generation sequencing technologies, with each sample representing a different bacterial species, strain or isolate. The obtained reads are assembled, generating a set of contigs for each sample. This set of partially assembled genomes is then analyzed using bioinformatics tools to gain insights into the bacterial evolutionary dynamics and genomic composition of these samples. Typical research challenges are: (i) inferring the core genome and pangenome (the set of genes shared by all members of the analyzed clade and the set of genes shared by at least one member of the analyzed clade, respectively) (1); (ii) reconstructing the evolutionary history of the analyzed samples as a phylogenetic tree (2); (iii) analyzing the variation in GC content among samples (3); (iv) analyzing the gene gain and loss dynamics, which is often an indication of the intensity of horizontal gene transfer (4); (v) detecting genes that are likely to have experienced positive selection (5–7).

The above computations require the use of multiple bioinformatics tools and ad-hoc programming scripts to handle information flow among the various programs, which in turn necessitates a dedicated bioinformatician to conduct such analyses. As a result, research laboratories began implementing their own in-house analysis pipelines, and later, different analysis applications began to emerge (8–10). These applications require specific working environments (i.e., operating systems), computation power (multicore machines), and more than basic technological skills (e.g., installation and running). Previously developed web tools to analyze sequenced microbial genomes are the MG-RAST, Pan-X and PGAweb web servers. MG-RAST allows finding and annotating gene functions or pathways by comparing genes to other databases (11). It differs from M1CR0B1AL1Z3R in that the latter focuses on comparing genomes rather than on their annotation and does not rely on external databases. The Pan-X web server provides ready-made examples of different microbial datasets (8). However, this web server does not allow providing unpublished genomic sequences as input. PGAweb provides several outputs, such as an analysis of the orthologous groups and reconstruction of the phylogenetic relationships among the sequences (12). However, in contrast to the M1CR0B1AL1Z3R web server, described below, it can only handle up to 50 genomic samples. In addition, phylogenetic relationships are reconstructed using neighbor joining or UPGMA, which are known to be less accurate than state-of-the-art methodologies for tree reconstruction such as maximum-likelihood and Bayesian approaches (13).

**Figure 1.**
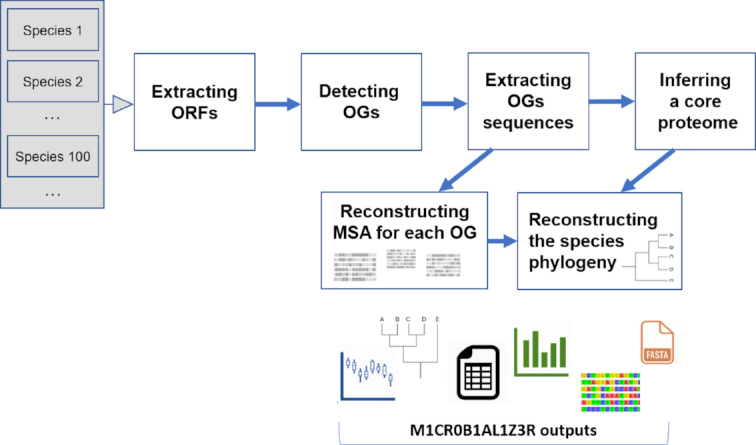
M1CR0B1AL1Z3R web server workflow. MSA, multiple sequence alignment. OG, orthologous group.

Here we present the M1CR0B1AL1Z3R (pronounced: microbializer) web server. M1CR0B1AL1Z3R was developed to facilitate microbial analyses and make them more accessible to the scientific community. M1CR0B1AL1Z3R utilizes a versatile computational pipeline that runs on the cloud and provides quick and easy analyses of bacterial genomics data for all users (Figure 1). No installation and no other prerequisites are needed. Visual and textual results that are ready for publication or further analysis are given as output.

## MATERIALS AND METHODS

### Input

The M1CR0B1AL1Z3R web server requires assembled genomic sequences (fully assembled or as contigs) from several clades. Each clade can represent a bacterial (or archaeal) isolate, strain or species. Each clade should be in a separate Fasta format file (such files are generated using assembly programs such as Velvet (14) or Canu (15)). Notably, in many metagenomic studies, the assignment of the various contigs to separate clades is unknown, and in this case, the data should be binned prior to running M1CR0B1AL1Z3R (16). To upload the files to M1CR0B1AL1Z3R, we ask the user to put them in a zipped folder (zip or tar.gz). Upon completion of the analyses, a link to the results is sent to the user if they choose to provide their email address. The results remain available on the web server for at least 3 months.

### Extracting putative open reading frames (ORFs)

We extract ORFs from each genome using Prodigal (17) in ‘normal’ mode. Prodigal uses an unsupervised machine learning approach to extract protein-coding ORFs.

### Extracting orthologous sets

A homology search is conducted in which each ORF is queried against all other ORFs in the database (all-against-all). Homology searches are executed using the equivalent of tBlastX in the MMSEQS2 program, which is ∼400 times faster than BLAST with similar accuracy (18). For each ORF, we record the top hit in each other genome. If ORF *x* in genome *i* is the top hit for ORF *y* in genome *j* and vice versa, these two ORFs are considered putative orthologs (best reciprocal hit, as in (19)). This pairwise analysis induces a graph in which each node is an ORF, and two nodes are connected if they are best reciprocal hits. An orthologous group is a set of nodes that are highly connected to each other and are separated from the rest of the nodes. We use the Markov Cluster (MCL) algorithm (as done in the OrthoMCL pipeline (20)) with default parameters (inflation parameter = 2.0) to detect these high-confidence orthologous groups.

### Multiple sequence alignments (MSAs) and phylogenetic tree reconstruction

For each orthologous group, all sequences are first translated and the resulting protein sequences are then aligned using MAFFT, with the ‘--auto’ flag, which automatically selects an appropriate MAFFT algorithm (L-INS-i, FFT-NS-i or FFT-NS-2) according to the size of the analyzed dataset (21). Sequences are then reverse-translated so that codon-level alignments can also be computed (as in (22)). A maximum-likelihood phylogenetic tree is reconstructed based on the concatenated protein MSA of all core genes, i.e., genes shared among all strains (see below), using RAxML (23) with default parameters, the LG replacement matrix (24), and a discrete gamma distribution with four categories and an invariant category (LG+G+I) to account for among-site-rate variation (of note, we have recently shown that when searching for the maximum-likelihood tree topology, using LG+G+I provides results that are as accurate as when a model selection step is introduced, and the latter is therefore not mandatory (25)). The tree is visualized using PhyD3 (26).

### GC content

For each genome, the GC content is computed from the set of ORFs using an in-house Python script.

### Output

The following results are provided: (i) a text file with ORF counts per genome and its graphical representation as a violin plot; (ii) a curated file listing the orthologous sets and a histogram providing the distribution of set sizes; (iii) the unaligned sequences, the multiple sequence alignment at the protein level and the multiple sequence alignment at the codon level for each orthologous set. Both protein alignments and codon alignments are often used in downstream analyses, e.g., to find protein motifs (27) and to search for positive Darwinian selection (6), respectively. The unaligned sequences are also available if the user wishes, for example, to realign the sequences using another alignment program; (iv) a table in which each row is an orthologous group and each column is the set of genes of a specific sample (genome). The *i,j* entry contains the corresponding gene name of the *i*^th^ group and *j*^th^ sample, if such an entry exists (this is especially useful if the input includes at least one annotated genome). In addition, we provide a Fasta file with the phyletic pattern of all ortholog groups. Each record contains a sequence of ‘1’s and ‘0’s in the *i*^th^ place, depending whether it has a member gene in the *i*^th^ orthologous group or not, respectively (28). The generated phyletic pattern data (together with the species tree) can be further analyzed by the GLOOME web server (4), which allows inference of gene gain and loss rates, and ancestral reconstruction of these events along the species tree. In addition, we specifically provide a file with the list of ORFs shared by all samples, i.e., the orthologous group comprising the core proteome, and the concatenated protein alignment of this core proteome in Fasta format. The web server also provides means to extract the proteome shared by *x*% of the analyzed strains (where *x* = 100 is the default core proteome); (v) the phylogenetic species tree representing the evolutionary relationships between all samples, both as a text file in Newick format and using an online interactive visualizer; (vi) a text file with the GC content of each genome and a graphical representation using a violin plot.

### Implementation

M1CR0B1AL1Z3R is implemented in Python 3.6. The source code is available at: https://github.com/orenavram/microbializer. The web server jobs are processed on ProLiant XL170r Gen9 servers, equipped with 128 GB RAM and 28 CPU cores per node. The Gallery, Overview, and Frequently Asked Questions (FAQ) sections of the web server should help users get the most out of the web server. A running example (different from the case studies analyzed in the Gallery) is also provided.

## CASE STUDIES

**Figure 2.**
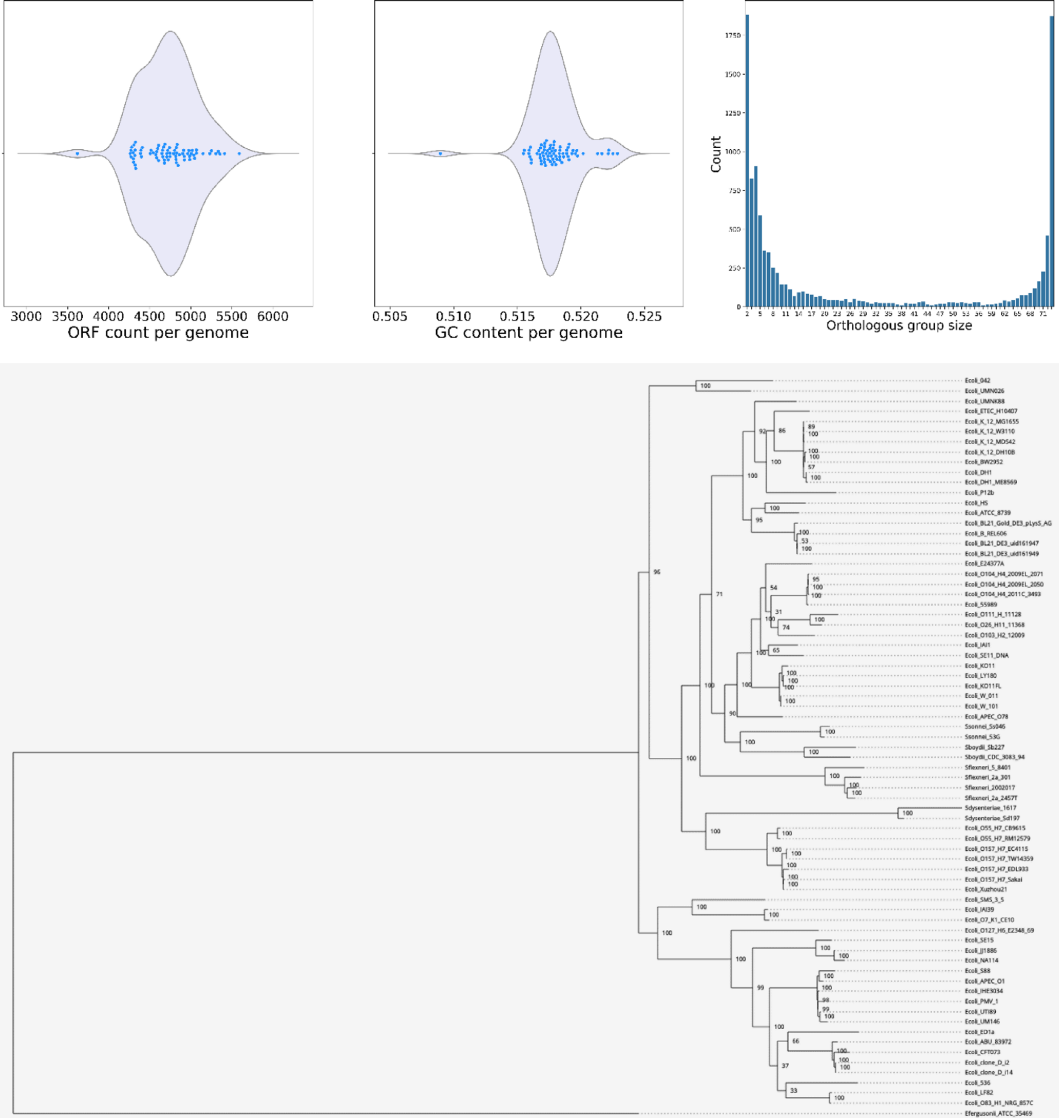
Selected visual outputs of the M1CR0B1AL1Z3R web server. Top panel (left to right): distribution of the number of ORFs in each genome; distribution of %GC in each genome; distribution of the sizes of the various orthologous groups. Bottom panel: phylogenetic tree representing the evolutionary relationships among all samples. The maximum-likelihood-based tree was reconstructed according to the core proteome as inferred from the orthologous group data.

The various analyses and outputs of M1CR0B1AL1Z3R are demonstrated using three datasets: (i) a set of 50 pathogenic *Escherichia coli* lineage ST131 genomes (29). This dataset represents highly similar clinical isolates of a specific bacterial species. We added an outgroup sequence to this dataset, the genomic sequence of *Escherichia fergusonii*; (ii) a collection of 73 different *Escherichia* genomes (72 of which are *E. coli* and one *E. fergusonii*). The 72 genomes are all fully sequenced *E. coli* genomes available as of December 2018 in the NCBI repository, and the *E. fergusonii* genome is used as an outgroup; (iii) a collection of 29 different Gammaproteobacteria genomes, taken from Pérez *et al.* (30). Together, these datasets demonstrate the applicability of M1CR0B1AL1Z3R for the analysis of a range of phylogenetic diversity, from different isolates to different species belonging to different bacterial orders. The complete results for these three examples are available in the Gallery section of the web server. For example, for dataset (ii), the number of ORFs varies from 3,621 to 5,592, with the smallest genome being 3,976,195 bp and the largest 5,697,240 bp. The entire set is comprised of 8,811 orthologous groups, 1,863 of which comprise the core genome. The multiple sequence alignment of the core proteome (618,921 amino acid sites) was used to reconstruct the maximum-likelihood phylogenetic tree, which is consistent with previously established *E. coli* phylogeny (31). The GC content of the analyzed genomes varies from 50.9 to 52.3%. The graphical outputs describing the ORF counts, orthologous group size dispersion, GC-content variation and phylogenetic relationships are shown in Figure 2.
